# Influence of Certain Medicinal Agents on the Bacillus of Tubercle in Man

**Published:** 1889-04

**Authors:** 


### Influence of Certain Medicinal Agents on the Bacillus of Tubercle in Man.

Dr. Hunter Mackenzie contributes a paper on the influence of certain medicinal agents upon the bacillus or tubercle in man, as a communication to the British Laryngological Association. He discusses the exact relation of the presence of the tubercle-bacillus to the disease in the lungs or larynx, and shows that the micro-organism may be present in the sputa of persons for several years without any definite signs of phthisis making their appearance. Although indicative of the tubercular changes in the tissues from whence they are derived, the bacilli give no indication as to the quiescence or activity of those changes. Nor do the numbers of the bacilli found in any given case indicate the extent or degree of the disease. In the larynx, however, the bacilli are rarely met with except in cases of definite and progressive changes, but it is doubtful whether they are derived from the purulent sputa from the diseased lungs, since they may occur with very little evidence of lung-mischief, and are often absent when grave degeneration is going on in the lung. Of the various methods which have been tried for the annihilation of the bacillus, Dr. Mackenzie speaks with impartial scepticism. That climatic influences have no destructive action upon the bacilli has been proved again and again. The climates of low temperature, and with a minimum of variation, would seem to promise the best, but results even in such climates as these are by no means conclusive that any positive action is exerted. Even less can be said of the action of general drugs. Local treatment with a view of destroying the vitality of the micro-organism has not been successful hitherto, nor is it likely that any measure of success will be obtained until an agent can be found which shall act at once actively on the bacillus and passively upon the tissues in which it exists. The bacillus itself is most difficult to influence even when isolated, and is only to be completely annihilated by means which are inapplicable within the human body. Iodoform, corrosive sublimate, and a host of other germicide agents have been employed locally in laryngeal tubercle without any satisfactory result.—*London Medical Recorder.*

